# A single session of a beach volleyball exergame did not improve state anxiety level in healthy adult women

**DOI:** 10.1186/s13102-024-00859-9

**Published:** 2024-03-11

**Authors:** Vinnycius Nunes de Oliveira, Ricardo Borges Viana, João Victor Rosa de Freitas, Mila Alves Matos Rodrigues, Marilia Santos Andrade, Rodrigo Luiz Vancini, Katja Weiss, Beat Knechtle, Claudio Andre Barbosa de Lira

**Affiliations:** 1 Faculdade de Educação Física e Dança, Universidade Federal de Goiás, Goiânia, Brazil; 2 Instituto de Educação Física e Esportes, Universidade Federal do Ceará, Fortaleza, Brazil; 3 Departamento de Fisiologia, Universidade Federal de São Paulo, São Paulo, Brazil; 4 Centro de Educação Física e Desportos, Universidade Federal do Espírito Santo, Vitória, Brazil; 5 Medbase St. Gallen Am Vadianplatz, Vadianstrasse 26, St. Gallen, 9001 Switzerland; 6 Institute of Primary Care, University of Zurich, Zurich, Switzerland

**Keywords:** Exergaming, Beach volleyball, Physical exercise, Feeling, Mental health, Anxiousness

## Abstract

This study evaluated the acute effect of the exergame Kinect Sports^®^ beach volleyball on state anxiety level in adult women. Thirty healthy adult women (age: 21 [4] years, body mass: 54.70 [19.50] kg, height: 1.61 ± 0.05 m, and body mass index: 21.87 [5.76] kg/m^2^, data are expressed as median [interquartile range] and as the mean ± standard deviation) were assigned to play an exergame of beach volleyball in singleplayer mode session (intervention session) for ~ 30 min using the Xbox 360 Kinect^®^ or remained seated (control session). State anxiety was evaluated before and after the intervention and control sessions through the State-Trait Anxiety Inventory. State anxiety obtained in both sessions (exergame and control) was classified as intermediate before (median: 36.00 [IQR: 4.75] and mean = 38.73 ± 7.23, respectively) and after (mean: 34.86 ± 6.81 and mean: 37.66 ± 8.44, respectively). Friedman test found no time significant effect on state anxiety of the sessions (χ^2^ [3] = 6.45, p-value = 0.092, Kendall’s W = 0.07 “trivial”). In conclusion, the present study showed that there were no significant differences in the state anxiety level after an acute session of the exergame beach volleyball.

## Key points

• Exergame beach volleyball presented high enjoyment and a moderate to strong future engagement possibility.

• A session of the volleyball exergame did not improve affectivity and state anxiety level compared with control condition.

• A session of the volleyball exergame elicited moderate physical exercise intensity.

## Introduction

Anxiety disorders are one of the most common psychiatric conditions, with an estimated 264 million people with anxiety disorders worldwide in 2015, corresponding to 3.6% of the population [1]. Brazil, in 2015, presented the highest prevalence of anxiety disorders in the world, corresponding to approximately 19 million individuals (9.3% of the Brazilian population) [1]. Anxiety is defined as a state of excessive concern, anticipation of future, and panic [2, 3] and has two classifications: state anxiety (transitory feelings, acute) and trait anxiety (usually feelings, chronic) [4, 5].

A mild and infrequent level of anxiety is a normal emotion during life because any individual worries about aspects such as personal or family problems, health, and money [6] that do not meet duration or intensity criteria to enable a clinical diagnosis [7]. In this latter case, symptoms of anxiety can impair quality of life and daily activities, such as work performance, relationships, and schoolwork [8]. Thus, for an individual with an anxiety disorder, the symptoms do not go away and can worsen over time [6]. Therefore, the study of therapeutic tools that might help to prevent and treat anxiety disorders is necessary.

In this context, physical exercise can be an important nonpharmacological tool to prevent and treat various outcomes, such as anxiety and sleep [9–12]. Conversely, physical inactivity is associated with the development of mental illness [9–11]. Indeed, previous studies that investigated acute and chronic effects of physical exercise showed improvements on anxiety symptoms [9, 11, 13–17]. However, most of the population stays physically inactive [18–21]. Guthold et al. evaluated data from nearly two million participants and showed that globally, in 2016, more than a quarter of all adults were not getting enough physical activity [20]. The main reasons presented by the population are lack of time, lack of company, lack of adequate climate, fear of injury, fear of leaving home unaccompanied, lack of motivation, physical tiredness, and lack of enjoyment [22–26].

To overcome these barriers, a new modality of physical exercise emerged, named exergames. Exergame is a modality of active videogame combining exercise and videogame [27–29] and can be used in home. The literature has shown that exergames may be more enjoyable than traditional programs of physical exercise [16, 30]. Exergames can simulate various types of physical exercise, for example, walking/running, sports (e.g., beach volleyball, basketball, bowling, yoga, tennis, table tennis, boxing), calisthenics exercises, and dance [16, 17, 31–33]. In addition, many studies have shown that exergames [16, 17, 33] elicit physiological responses classifying physical exercise as a moderate intensity according to the guidelines of the American College of Sports Medicine (ACSM) [34]. Therefore, it is reasonable to assume that exergames can be a useful tool to manage symptoms of anxiety.

Previous studies investigated the acute effects of exergame on state anxiety, and the results were not conclusive. Viana et al. evaluated the acute effect on state anxiety and enjoyment of a session of the exergame Zumba^®^ Fitness in the Xbox 360 Kinect^®^ of moderate intensity in singleplayer mode in young women and found a decrease in state anxiety after session and a high enjoyment level [17]. Morais et al. also evaluated the effect of the exergame Zumba^®^ Fitness in the Xbox 360 Kinect^®^ on state anxiety and enjoyment by comparing state anxiety level after the dance exergame session with that after a traditional aerobic exercise (walking/running on a treadmill). The authors found a reduction in state anxiety only after the dance exergame session [35]. Conversely, da Silva et al. compared the effect of the exergame Hollywood Workout in the Xbox 360 Kinect^®^ with a traditional calisthenics session on state anxiety level in healthy adult men and found no decrease in state anxiety after both sessions [33].

Notably, the main limitation of these studies was the lack of a non-exercise control group. To the best of our knowledge, no previous study has evaluate the acute effect of an exergame session on state anxiety and included a non-exercise control session in adult women. Recently, Viana et al. conducted a systematic review and meta-analysis to investigate the effects of exergame on anxiety level and found that although exergame interventions resulted in improvements in anxiety levels, they were not superior to the effects from non-exercise control interventions. According to the authors, the existing studies have small sample sizes, different research designs, and different populations [36]. Therefore, further studies are warranted to investigate the acute effects of exergames on state anxiety level.

Thus, the present study aimed to evaluate the acute effect of the exergame Kinect Sports^®^ beach volleyball on state anxiety in healthy adult women. The secondary aims were to assess affectivity, enjoyment, and exercise intensity elicited by the exergame beach volleyball session. Considering that previous studies showed a decrease in state anxiety in adult women after one session of dance exergame, we hypothesized that one session of the exergame Kinect Sports^®^ beach volleyball in single-player mode would also provide a decrease in state anxiety in healthy adult women [16, 36].

## Materials and methods

### Participants

A posteriori power analysis was performed using G*Power (version 3.1.9.7; Frans Faul, University of Kiel, Germany) [37]. Based on a moderate correlation between repeated measurements (*r* = 0.5) and a partial eta squared (η^2^_p_) of 0.29 (converted to effect size *f *of 0.639), the sample size of 30 participants promoted a statistical power of ≥ 95%.

The participants were recruited through social media (Instagram^®^ and WhatsApp^®^), direct contact, and announcements on institutional websites. Thirty-four healthy adult women participated in the study (a convenience sample) (Table 1). The inclusion criteria were (i) female sex and (ii) aged between 18 and 40 years. The exclusion criteria were (i) contraindications to physical activity (assessed using the Physical Activity Readiness Questionnaire); (ii) diagnosis of mood and/or anxiety disorders; (iii) use of stimulants (e.g., psychotropic drugs); and (iv) being in the menstrual period.

Four participants were excluded from the study because they had an illness with repercussions on mood. The participants were not clinically diagnosed with anxiety, depression, and/or other mental disorders according to a self-report made by each participant. The literature show that anxiety symptoms do not only appear in people with anxiety and can also affect non-clinical populations, impairing quality of life and daily activities, such as work performance, relationships, and schoolwork. In this sense, strategies for managing these symptoms are desirable. Furthermore, although the effect of exercise on anxiety is well established, a large part of the population is physically inactive. Therefore, exercise programs considered more fun such as exergame should be investigated.

**Fig. 1:**
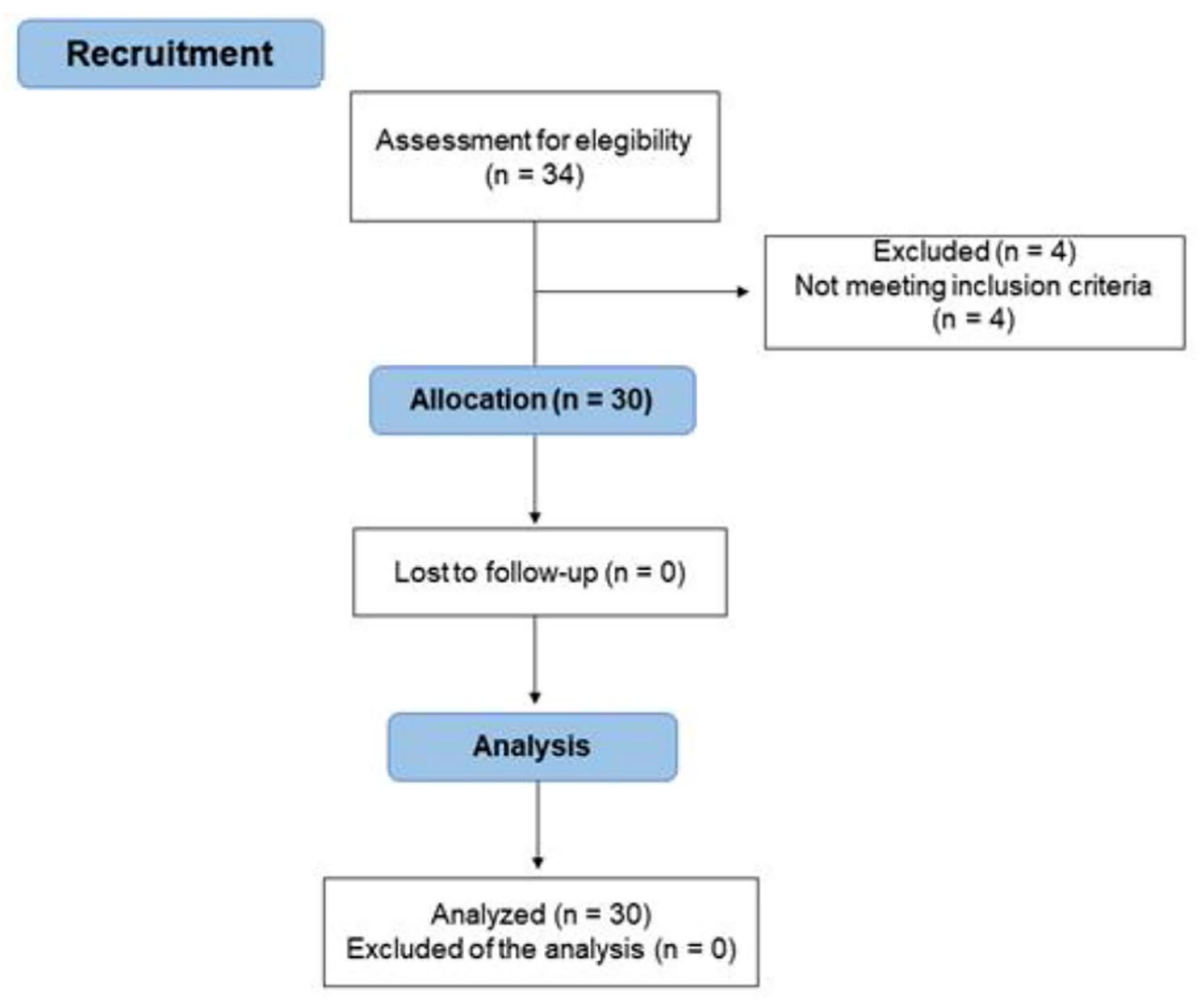
Diagram flow of the study

In this study, we evaluated only young adult women because the literature showed that anxiety is more prevalent in this population than in men [1]. Informed consent was obtained from all participants included in this study. All experimental procedures were approved by the Research Ethics Committee of the Federal University of Goiás (approval number: 01970818.8.0000.5083) and followed the principles outlined in the Declaration of Helsinki. The flow diagram of the study is presented in Fig. 1.

**Table 1:** Characteristics of the participants (*n* = 30)

Variables	Median [IQR]^a^	Min-Max^d^
Age (years)	21 [4]	18–32
Body Mass (kg)	54.70 [19.50]	40.40-114.90
Height (m)^c^	1.61 ± 0.05	1.50–1.72
Body Mass Index (kg/m²)	21.87 [5.76]	16.18-40.00
Trait anxiety (20–80)^c^	42.63 ± 8.29	30–62
Estimated $\dot{V}$O_2_max (ml/kg/min)^b,c^	32.90 ± 6.38	19.73-48.00

^a^IQR: interquartile range; ^b^$\dot{V}$O_2_max: maximum oxygen uptake. ^c^Data are presented as the mean ± standard deviation, because they were normally distributed. ^d^Min-Max: minimum and maximum values

### Study design

This was an experimental within-participants study composed of three visits. At the first visit, the participants were submitted to anamnesis, anxiety trait assessment, anthropometric assessment, cardiorespiratory fitness assessment, and randomization of subsequent visits. In addition, all participants were familiarized with a match of the exergame beach volleyball for approximately six minutes. At the second and third visits, the participants played a session of the exergame beach volleyball in singleplayer mode (intervention) or remained seated (control session) for 30 min, and the order depended on randomization. During and at the end of each match, heart rate (HR) and rating of perceived exertion (RPE) were measured to characterize the session exercise intensity. State anxiety and affectivity level were assessed before and after the exergame and control sessions. Furthermore, enjoyment and future engagement possibility were evaluated only after the exergame session. In order to avoid bias, the scales and questionnaires were applied by an experienced researcher with these tools. During data collection, the researcher did not express facial or behavioral reactions to the participants’ responses.

### Experimental procedures

#### Anamnesis and anthropometric assessment

The anamnesis was performed through the Physical Activity Readiness Questionnaire (PAR-Q). The PAR-Q contains seven questions to evaluate the general health condition of the participants and whether they were fit to perform exercise. If a participant answered “yes” to one or more questions, the participant was excluded from the study [38]. Body mass was measured using a digital balance (Omron, HN-289, USA) to the nearest 0.1 kg, and body height was measured using a wall stadiometer (Caumaq, Brazil) to the nearest 0.1 cm. Thereafter, body mass index was calculated by dividing body mass by body height squared (kg/m²).

#### Cardiorespiratory fitness assessment

Cardiorespiratory fitness of the participants was assessed through the Ebbeling test, performed on a motorized treadmill (ATL, Inbramed, Brazil). This test was chosen because of its easy application, low cost and provides a valid and time-efficient method for estimating maximum oxygen uptake (r² = 0.92) [39]. The Ebbeling protocol is composed of two four-minute stages [39]. During the first four minutes of the protocol, participants walked or ran at a 0% slope with a speed corresponding to an HR range from 50 to 70% of the maximal HR (HRmax) predicted for age. Finally, for the last four minutes of the protocol, the treadmill slope increased to 5% while the speed remained the same [39]. Thereafter, HR was measured at the end of the protocol. After the protocol, the predicted maximal oxygen uptake ($\dot{V}{O}_{2}max$) was estimated through the equation below:

$$\dot{V}{O}_{2}max = 15.1 + 21.8\,(speed) - 0.327\,(HR) - 0.263$$

$$\,(speed \times age) + 0.00504\,(HR \times age) + 5.98 \times sex.$$

Where, speed was expressed in miles per hour, HR in beats per minute (bpm), age in years and sex was 0 for women or 1 for men.

#### Exergame and control session

The console used in this study was the Xbox 360 (Microsoft^®^, USA). Xbox has a movement sensor, Kinect^®^ (Microsoft, USA). This sensor allows players to interact with videogames without the necessity of remote control. The exergame Kinect Sports^®^ included six modalities of sports (i.e., beach volleyball, soccer, track and field, bowling, boxing, and table tennis). In the current study, participants played beach volleyball, because several muscles are recruited to maintain a player’s performance during match [40]. This exergame is inherently competitive with a possible win or lose outcome. Each visit was performed within an interval of 24–72 h (wash-out period). The participants were instructed to visit the laboratory wearing appropriate clothes to perform physical exercise, to refrain from eating two hours before exercising, and to abstain from caffeine, alcohol, and strenuous physical activity on the day of the experiment. The temperature in the laboratory ranged from 21 to 23 °C. Each participant was supervised by an experienced researcher. Furthermore, conversation was minimized during all data collection periods, and the presence of people was restricted only to researchers and the participant involved in the study. During the control session, the participants remained seated for 30 min. The participants were not allowed to read, study, listen to music, or use their smartphones during the sessions. The exergame session lasted approximately 30 min and the number of sets was variable according to participants to reach the same time of control session.

#### State-trait anxiety assessment

Anxiety of the participants was assessed through the state component from the State-Trait Anxiety Inventory (STAI), an instrument already translated and validated for Brazilian Portuguese [5, 41]. Briefly, the STAI is a 40-item self-reported assessment scale made up of two 20-item anxiety subscales (state and trait). The state subscale describes an individual’s feelings at a particular time, whereas the trait subscale describes an individual’s usual feelings. Each item of the STAI is given a score of 1 to 4. Overall scores can range from a minimum of 20 to a maximum of 80. A score equal to or lower than 30 indicates a low level of state anxiety, a score ranging from 31 to 49 indicates an intermediate level of state anxiety, and a score higher than or equal to 50 indicates a high level of state anxiety [41]. The STAI was answered by participants inside a sound-attenuated room. The STAI was chosen because of its easy application and low cost.

#### Physical exercise intensity assessment

Physical exercise intensity was monitored by measuring participants’ HR and RPE. HR was monitored using an HR monitor (H10, Polar, Finland). The HRmax was estimated using the following age-predicted HRmax equation: HRmax = 208 − 0.7 × age [42]. RPE was monitored using the Borg Scale (6–20) [43]. The classification of the exercise intensity followed the criterion adopted by the ACSM [34].

#### Enjoyment assessment

Enjoyment was assessed through the Physical Activity Enjoyment Scale, a scale translated and validated for Brazilian Portuguese [44]. This scale has 18 items, and each item has two opposite poles that are separated by a 7-point Likert scale (1 = “I like”; 7 = “I hate”; 4 = “neutral”). Scores range from a minimum of 18 (no enjoyment at all) to a maximum of 126 (highest enjoyment level). The scale was applied only after the exergame session [44].

#### Future engagement and affectivity assessment

Affectivity was assessed through the feeling scale [44]. This scale ranges from − 5 to + 5 (-5 = very bad, -3 = poorly, -1 = reasonably bad, 0 = neutral, 1 = reasonably well, 3 = well, and 5 = very well). This scale was applied before and after the exergame and control session [44]. Future engagement was assessed after sessions through a scale adapted by Focht et al. [45]. The scale ranges from 0 to 100%, in which verbal anchors are provided at points 0%, 50% and 100%, where 0% means no intention, 50% means moderate intention, and 100% means strong intention. These tools were already used in previous studies [33, 45–49].

### Statistical analysis

The Shapiro‒Wilk test was used to test data normality. As the state anxiety and affectivity data did not present a normal distribution, the Friedman test was used to evaluate state anxiety level and affectivity level between times (pre-exergame session, post-exergame session, pre-control session, and post-control session). When necessary, Conover’s post hoc test was used to identify the differences between times. Kendall’s W was used as the effect size for the Friedman test. Kendall’s W values were classified according to Cohen’s *d* as “trivial” (< 0.10), “small” (0.10 ≤ to < 0.30), “medium” (0.30 ≤ to < 0.50), and “large” (≥ 0.5) [50]. The variables age, body mass, body mass index, future engagement possibility, RPE, number of matches, and game time presented a non-normal distribution. However, body height, trait anxiety, $\dot{V}$O_2_max, enjoyment level, mean HR, percentage of HRmax, number of wins, state anxiety after exergame, and state anxiety before and after the control session presented normal distributions. Parametric data are presented as the mean ± standard deviation, and non-parametric data are presented as the median and interquartile range (IQR). All data were analyzed through Jeffrey’s Amazing Statistics Program (JASP, version 0.16.4, University of Amsterdam, Netherlands). The level of significance assumed was α < 0.05.

## Results

There were no medical intercurrences during the study. The median number of matches played by the participants was 6 [IQR: 1], while the mean number of wins was 4.73 ± 2.10. The median game time reached by the participants was 29.47 [IQR: 2.30] minutes.

### State anxiety level

**Fig. 2:**
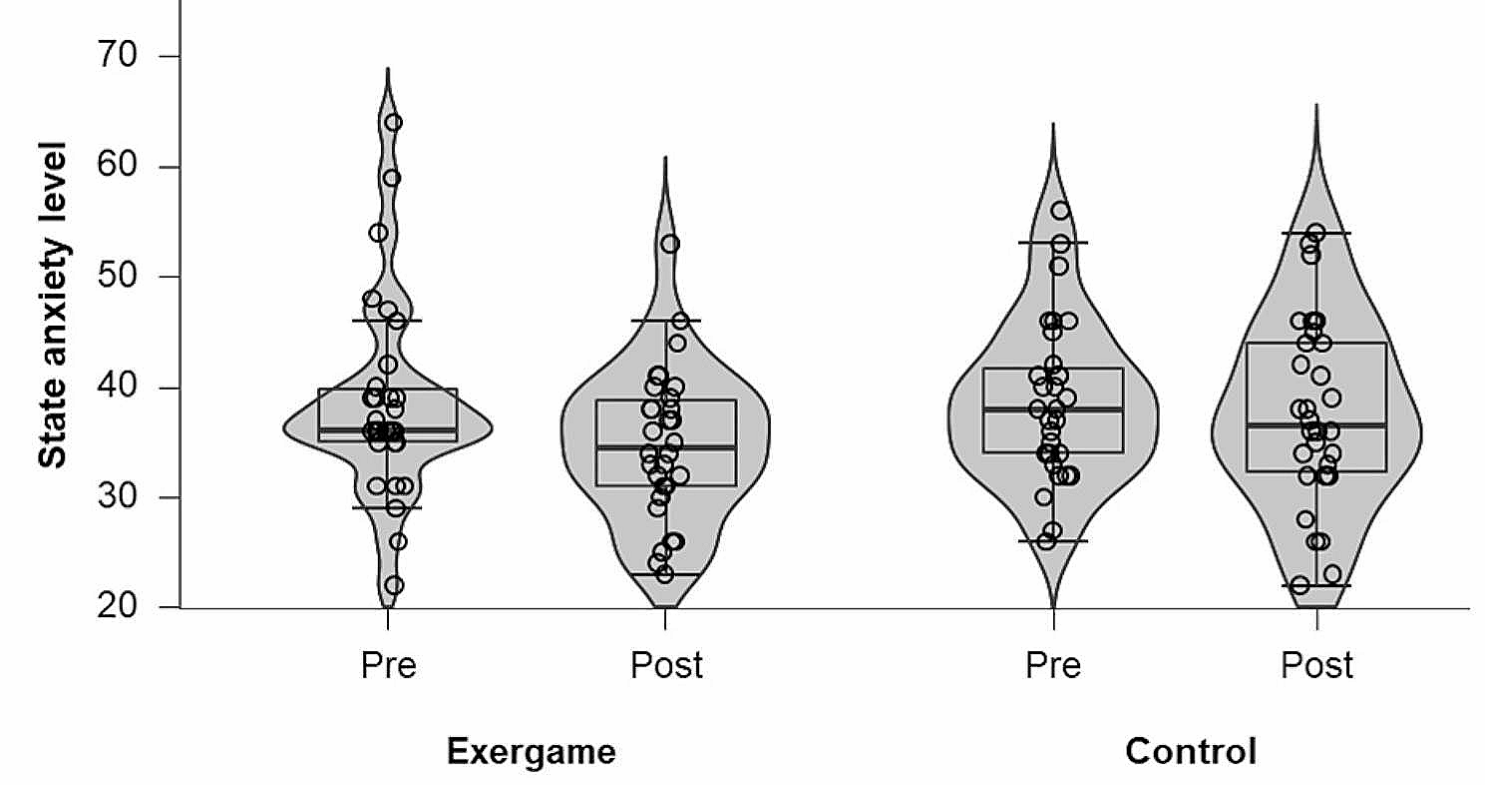
State anxiety before and after a session of the exergame beach volleyball in single player mode and control session. There was no significant difference between times (*P*-value = 0.092)

State anxiety obtained in both sessions (exergame and control) was classified as intermediate before (median: 36.00 [IQR: 4.75] and mean = 38.73 ± 7.23, respectively) and after (mean: 34.86 ± 6.81 and mean: 37.66 ± 8.44, respectively) sessions. Friedman test found no significant time effect on state anxiety of the sessions (χ^2^ [3] = 6.45, p-value = 0.092, Kendall’s W = 0.07 “trivial”) (Fig. 2).

### Affectivity level

**Fig. 3:**
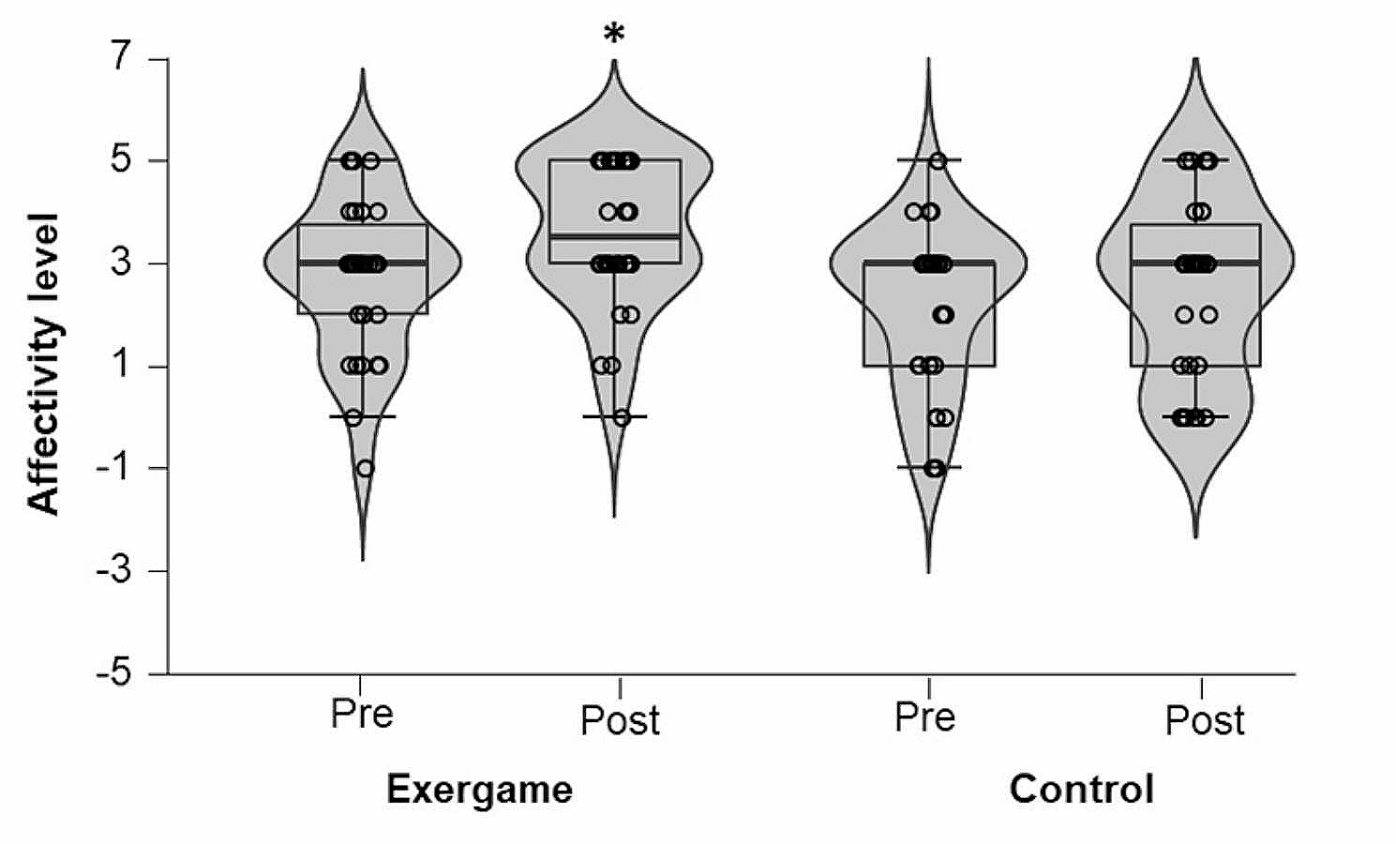
Affectivity level before and after a session of the exergame beach volleyball in single player mode and a non-exercise control session. There was a significant difference between times (*P*-value < 0.001). *Significant difference compared to the time pre (exergame)

The affectivity level reported by the participants in both sessions (exergame and control) was classified as “well” before (median: 3.00 [IQR: 1.75] and median = 3.00 [IQR: 2.00], respectively) and after (median: 3.50 [IQR: 2.00] and median: 3.00 [IQR: 2.75], respectively). The Friedman test found a significant time effect on the affectivity of the participants (χ^2^ [3] = 22.54, p-value < 0.001, Kendall’s W = 0.25 “small”). Conover’s post hoc test adjusted by Bonferroni’s correction showed that affectivity after the exergame session was larger than that measured before the exergame session (p-value = 0.005). However, Conover’s post hoc test adjusted by Bonferroni’s correction showed no significant difference in affectivity between the post-exergame and post-control session times (p-value = 0.067) (Fig. 3).

### Enjoyment and future engagement possibility

The participants reported a high enjoyment level after the exergame session (mean: 109.60 ± 11.72), corresponding to 87% of the maximal value provided by the enjoyment scale used. In relation to future engagement in physical exercise, the participants reported a strong to moderate intention for the exergame session (median: 75 [IQR: 30]).

### Exercise intensity

The mean HR presented by the participants during the exergame session was 133 ± 18 bpm, corresponding to 69 ± 9% of their HRmax. The median RPE reported by the participants during the exergame session was 12 [IQR: 5].

## Discussion

The present study evaluated the acute effect of a single session of the exergame Kinect Sports^®^ beach volleyball versus a non-exercise control session on state anxiety and affectivity in healthy adult women. Additionally, the enjoyment, future engagement, and exercise intensity of the exergame Kinect Sports^®^ beach volleyball were evaluated. Our initial hypothesis was that the exergame Kinect Sports^®^ beach volleyball would provide a decrease in state anxiety. However, contrary to our initial hypothesis, a single session of the exergame Kinect Sports^®^ Beach Volleyball did not improve state anxiety level in healthy adult women. Therefore, our results did not confirm our initial hypothesis.

Viana et al. and Morais et al. evaluated the acute effect on state anxiety and enjoyment of a session of the exergame Zumba^®^ Fitness in the Xbox 360 Kinect^®^ in healthy young women. Both studies found reductions in state anxiety after an exergame session [17, 35]. This fact has been common in the literature on exergames and anxiety; in other words, previous studies found significant improvements in anxiety level, however, it was not superior to the effect from non-exercise control sessions [36]. In the present study, we found no differences in state anxiety between exergame and non-exercise control sessions. Our results may be influenced by the type of exergame investigated since we used a beach volleyball exergame, while Viana et al. and Morais et al. used a dance exergame [17, 35]. We expanded the findings of Viana et al. and Morais et al. since we included a non-exercise control session and compared it with an exergame session [17, 35].

Recently, de Oliveira et al. evaluated the acute effect of the exergame Kinect Sports^®^ beach volleyball in the Xbox 360 Kinect^®^ in singleplayer mode on the state anxiety of adult men, and the authors found no significant decreases in state anxiety after an exergame session [32]. da Silva et al. compared the acute effects of an exergame-based calisthenics session versus a traditional calisthenics session on anxiety level in healthy adult men [33]. The authors did not find significant differences between the sessions. In the present study, we also found no significant reduction in state anxiety level after an exergame beach volleyball session. Thus, our findings were similar to previous studies [32, 33]. The literature has frequently shown that moderate-intensity physical exercise and high enjoyment level after physical exercise can be important factors in improving anxiety level [16, 17, 51, 52, 21, 53–59]. In the present study, exercise intensity was classified as moderate, according to HR and RPE obtained during the exergame session, and the participants reported a high enjoyment level after an exergame session [34]. Our findings add to the literature that moderate intensity physical exercise (performed through the exergame Kinect Sports^®^ beach volleyball) and high enjoyment level seem to be insufficient to improve state anxiety in healthy adult women. Therefore, the decrease in anxiety seems to be related to the type of exergame.

A bias that might influence the results from studies about anxiety is the recruitment of persons with average or lower levels of state anxiety; this situation is called the floor effect [9]. Thus, it would not be possible to decrease further anxiety level. Indeed, Knapen et al. showed that individuals with anxiety disorders show a larger decrease in state anxiety after physical exercise [60]. In the present study, participants were healthy women and presented intermediate anxiety level, considered normal state anxiety. This fact may have contributed to explaining the results that were found.

A secondary aim of our study was to evaluate the affectivity level of the exergame session. We found no significant differences in the affectivity level between exergame and non-exercise control sessions. Nevertheless, there was a significant improvement in affectivity level after the exergame session; however, it was no longer present when compared to a non-exercise control session. da Silva et al. compared the acute effects of an exergame-based calisthenics session versus a traditional calisthenics session on affectivity level in healthy adult men [33]. The results showed that the affectivity was classified as “well” after both sessions, with no significant differences between the sessions. Our results are in line with those reported by da Silva et al., since the affectivity level was similar between exergame and non-exercise control sessions [33]. The participants’ characteristics, self-efficacy, and tolerance to the type of exercise may explain the results found regarding the affectivity level [33, 61]. Our findings add that exergame beach volleyball is a modality that acutely did not improve affectivity level.

The present study also evaluated the enjoyment level and future engagement of the exergame session. Participants reported a high level of enjoyment (corresponding to 87% of the maximal possible score) and rated future engagement after the exergame session as strong to moderate intention. These results may be related to the fact that younger adult individuals are more receptive to innovative technology [18]. Physical exercises that provide a high level of enjoyment and future engagement can increase the constancy and adherence to physical exercise programs [22–26]. Indeed, a high enjoyment level has been common among various exergames [16, 17, 32, 33, 62]. Our findings add that exergame beach volleyball is a modality that presents high enjoyment and a strong to moderate engagement future.

Finally, as mentioned previously, exergame beach volleyball evoked a mean HR and RPE response corresponding to a moderate intensity physical exercise based on the criteria established by the ACSM. A previous study found a different exergame intensity (light intensity) using the same exergame protocol adopted in the present study [32]. These contradictory results might be related to the fact that those authors recruited healthy and physically active men ($\dot{V}$O_2_max ≅ 52 ml/kg/min), while we recruited only healthy women (estimated $\dot{V}$O_2_max ≅ 33 ml/kg/min) [32]. Thus, it is possible that the exergame beach volleyball from Kinect Sports^®^ did not challenge the participant’s cardiorespiratory fitness level enrolled in the previous study mentioned above [32]. Moreover, moderate intensity has been found in a dance-based exergame in adult women [16, 17]. Thus, according to our results, the exergame Kinect Sports^®^ beach volleyball can be prescribed as an alternative to healthy adult women who seek to reach the recommendation of the quantity of physical exercise proposed by the ACSM [34].

Our study is not without limitations. First, as we used questionnaires and scales, the results rely on the honesty of the participants. Second, we did not investigate a clinical population with a diagnosis of anxiety disorders. Third, this study investigated only healthy adult women and tested only a modality of the exergame Kinect Sports^®^; therefore, caution should be taken when extrapolating our results for other exergames. Fourth, we did not examine any endocrine response (e.g., increased norepinephrine, serotonin, and beta-endorphins and increased parasympathetic activity) and/or psychological mechanisms (e.g., increased self-efficacy, distraction, and a sense of mastery) that are responsible for reducing state anxiety. Nevertheless, we believe that these limitations do not prevent the conclusions of the present study from being drawn. Future studies should investigate the acute and chronic effects of the exergame Kinect Sports^®^ beach volleyball on state anxiety in individuals with anxiety disorders as well as include participants from both sexes and different ages to better elucidate this matter.

## Conclusions

The present study showed that there were no significant differences in the state anxiety level and affectivity level after an acute session of the exergame beach volleyball. However, the exergame beach volleyball elicited high enjoyment and presented a strong to moderate future engagement possibility.

## Data Availability

Available upon request (please contact Vinnycius Nunes de Oliveira, vinnyciusnunes@discente.ufg.br).
